# Hypopharyngeal Venous Malformation Mimicking Malignancy: A Case Report

**DOI:** 10.7759/cureus.110117

**Published:** 2026-06-02

**Authors:** Emma C Dunn, Spencer Short, Michael McGlone, Gary Stopyra, Jason Ohlstein

**Affiliations:** 1 Medicine, University of South Florida Morsani College of Medicine, Tampa, USA; 2 Otolaryngology - Head and Neck Surgery, Lehigh Valley Health Network, Allentown, USA; 3 Pathology, Lehigh Valley Health Network, Allentown, USA

**Keywords:** airway, dysphagia, hypopharynx, phlebolith, vascular malformation, venous malformation

## Abstract

Hypopharyngeal venous malformations are rare and may mimic malignancy on imaging, creating diagnostic and management challenges, particularly when tissue diagnosis is required.

A 61-year-old woman presented with progressive dysphagia and odynophagia. Contrast-enhanced computed tomography (CT) of the neck demonstrated an enhancing lesion involving the left tongue base, aryepiglottic fold, and hypopharynx, with associated coarse calcifications, raising concern for neoplasia. Flexible laryngoscopy revealed a compressible, purplish, vascular-appearing lesion. The patient underwent direct laryngoscopy with biopsy using a Lindholm laryngoscope with rigid telescope assistance, during which bleeding was successfully controlled with epinephrine-soaked pledgets. Histopathological analysis confirmed a venous malformation with phleboliths and features of a chronic low-flow vascular lesion, including positive immunohistochemical staining for CD34, CD31, and smooth muscle actin.

Hypopharyngeal venous malformations can closely resemble malignancy on imaging. Recognition of characteristic features, including calcifications and compressibility on endoscopy, is essential to guide diagnosis. This case demonstrates that biopsy can be performed safely in selected patients with appropriate airway planning and hemostatic precautions.

## Introduction

Vascular malformations of the hypopharynx are exceedingly rare, accounting for less than 1% of all vascular malformations [1]. Despite their rarity, they are clinically significant due to their potential to cause dysphagia, airway compromise, and diagnostic uncertainty, particularly when cross-sectional imaging findings mimic malignancy. Venous malformations are congenital slow-flow vascular lesions composed of dysplastic, thin-walled venous channels that often remain asymptomatic until adulthood, when progressive enlargement or thrombosis leads to symptoms such as dysphagia, globus sensation, or odynophagia [2]. On physical examination, these lesions are characteristically bluish and compressible, consistent with a low-flow vascular malformation, a feature that can help distinguish them from high-flow lesions and neoplasms.

Although 40%-60% of all vascular malformations in the body occur in the head and neck region, they most commonly involve the oral cavity and midface [3]. Involvement of the hypopharynx and supraglottis is distinctly uncommon and has been described primarily in isolated case reports [3,4]. The deep location of the hypopharynx, its proximity to critical airway structures (including the aryepiglottic folds, piriform sinuses, and laryngeal inlet), and the nonspecific presentation of these lesions can delay diagnosis and complicate management decisions.

We present a case of a large hypopharyngeal venous malformation in an adult patient to highlight the diagnostic challenges, the role of contrast-enhanced computed tomography (CT) in characterizing vascular lesions when magnetic resonance imaging (MRI) is unavailable, and the considerations for safe biopsy of vascular lesions in this anatomically complex region.

## Case presentation

A 61-year-old woman with a history of congestive heart failure, idiopathic cardiomyopathy, peripheral arterial disease, hypertension, dyslipidemia, aortic valve fibroblastoma, bladder tumor, and tobacco use disorder presented to the emergency department in February 2025 with progressive left-sided neck pain, dysphagia, and odynophagia. In October 2024, a polypoid lesion of the left arytenoid had been incidentally identified during intubation for an unrelated procedure. Over the subsequent months, she developed worsening dysphagia to solid foods and pills, requiring multiple swallowing attempts to tolerate food. She was able to tolerate liquids. She denied hoarseness, dyspnea, difficulty managing secretions, or hemoptysis.

Contrast-enhanced CT of the neck performed in the emergency department demonstrated a 3.5 × 1.0 cm markedly enhancing mass along the left tongue base, involving the left lingual tonsil and extending inferiorly into the hypopharynx, terminating just above the left vallecula (Figure 1). Adjacent enhancing nodules were noted anterior to the left vallecula, raising concern for local extension. Persistent thickening of the left aryepiglottic fold with superimposed nodular calcifications was identified, obliterating the left piriform sinus. These calcifications had been noted on a prior PET scan from 2021, suggesting a longstanding process. The overall appearance was considered possibly neoplastic. The patient was referred for otolaryngology evaluation. Flexible laryngoscopy demonstrated normal vocal cord mobility and a purplish, compressible, vascular-appearing lesion extending from the tongue base toward the left supraglottis.

**Figure 1:**
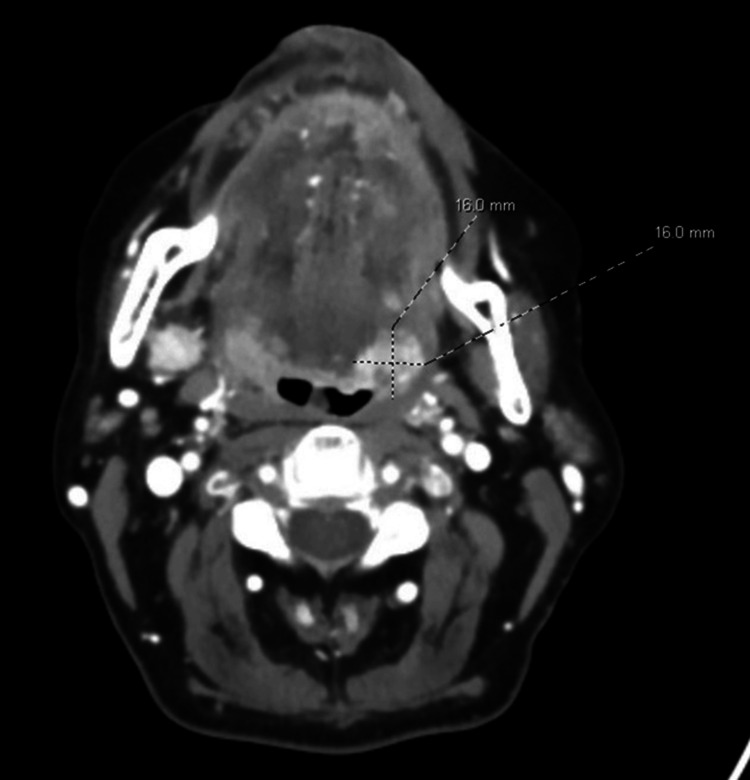
Contrast-enhanced computed tomography (CT) demonstrating an enhancing lesion involving the left tongue base and hypopharynx, with associated calcifications, suggestive of a vascular malformation.

At the three-month follow-up, the patient reported worsening dysphagia, odynophagia, globus sensation, intermittent hoarseness, and new headaches. Physical examination revealed a 1 cm varicosity of the left lateral posterior tongue. Flexible laryngoscopy demonstrated interval enlargement of the lesion involving the left aryepiglottic fold and tongue base, with preserved symmetric vocal fold mobility.

Repeat contrast-enhanced CT imaging in July 2025 demonstrated a 2.4 × 1.6 × 1.6 cm enhancing mass involving the left tongue base, glossotonsillar sulcus, and left lingual tonsil, with extension into the vallecula and piriform sinus. Coarse nodular calcifications were again noted in the bilateral paraglottic regions and left supraglottic soft tissues. Mild narrowing of the laryngeal inlet was present, likely due to a combination of soft tissue fullness and deglutition artifact. Preoperative flexible laryngoscopy at the bedside confirmed a purplish lesion adjacent to the left aryepiglottic fold and epiglottis (Figure 2).

**Figure 2:**
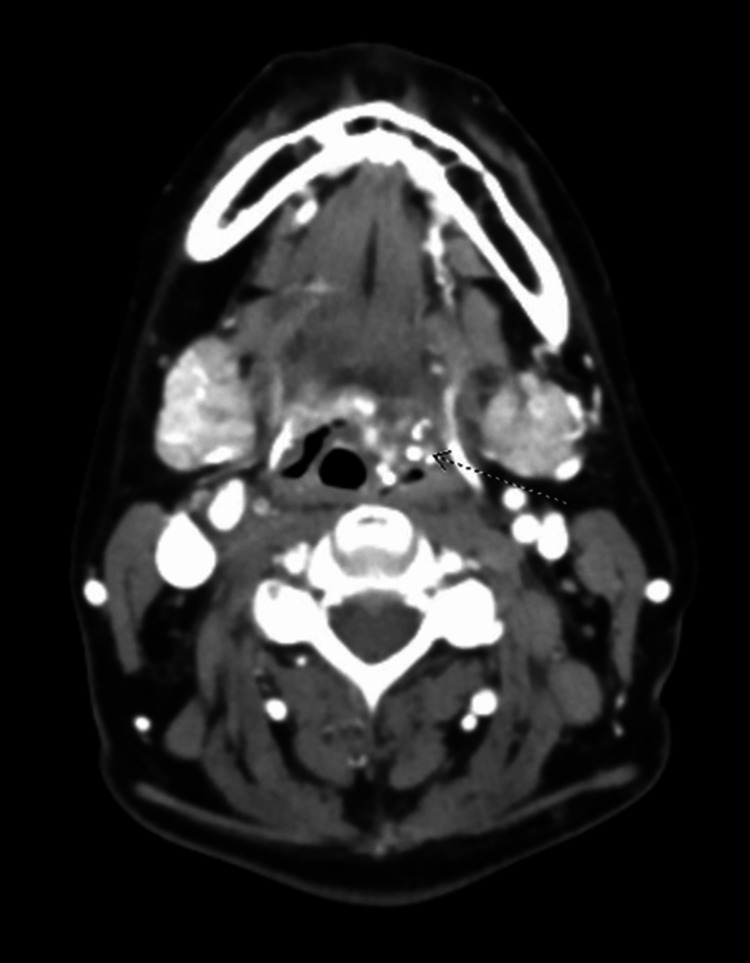
Axial computed tomography (CT) demonstrating the extent of the lesion within the hypopharynx, with associated airway narrowing.

MRI was not performed given the patient's implanted cardiac pacemaker device, and the lesion was presumed to represent a vascular malformation.

The patient underwent direct laryngoscopy with biopsy and concurrent rigid esophagoscopy under general anesthesia. A Lindholm laryngoscope was used with rigid telescope assistance for magnification. Intraoperative findings included a hypervascular lesion along the left supraglottis, measuring approximately 1.5 cm with associated calcification, and a nodular, firm lesion on the left tongue base measuring approximately 1 cm. Both sites were biopsied using cupped forceps and sent for permanent histopathology. Significant bleeding occurred and was controlled with epinephrine-soaked pledgets (Figure 3).

**Figure 3:**
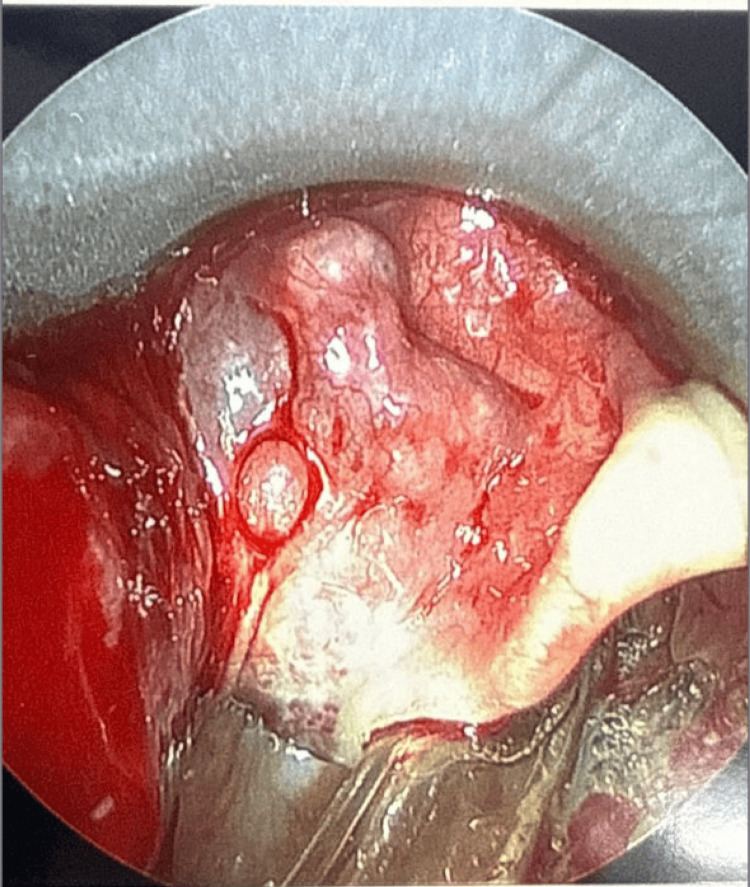
Intraoperative endoscopic view demonstrating a hypervascular lesion of the left supraglottis, with active bleeding during biopsy.

No additional abnormalities were identified on concurrent rigid esophagoscopy. There were no intraoperative or postoperative complications.

Histopathology of the left hypopharyngeal lesion and left base of the tongue confirmed a venous malformation involving the hypopharyngeal and base of tongue submucosa, underlying skeletal muscle, and adipose tissue. The lesion consisted of aggregates of disordered vascular structures of varying calibers, highlighted by immunohistochemical staining for vascular markers (CD34, CD31, smooth muscle actin). Elastin staining demonstrated irregular distribution of elastin fibers within the vessel walls. The lesion demonstrated intraluminal thrombi, chronic inflammation, hemosiderin deposition, and phleboliths, consistent with a longstanding, chronic low-flow lesion. No dysplasia of the overlying squamous mucosa or malignancy was identified. Congo red staining for amyloid was negative. Specimen dimensions were 1.0 × 0.4 × 0.3 cm and 1.0 × 0.5 × 0.4 cm for the left hypopharyngeal lesion and 1.0 × 0.8 × 0.4 cm for the left base of the tongue.

The patient was discharged on postoperative day 1 on a soft diet and resumed all home medications. At follow-up, approximately six weeks postoperatively, she was managed with analgesics (magic mouthwash) for symptomatic pain relief. Flexible endoscopy demonstrated a residual but non-obstructive vascular lesion extending from the tongue base along the aryepiglottic fold into the piriform sinus (Figure 4).

**Figure 4:**
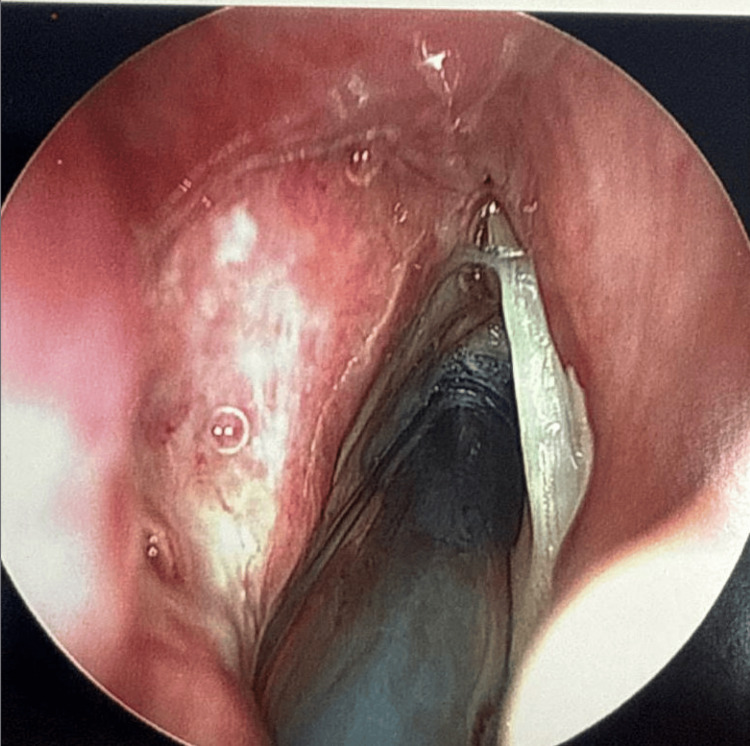
Endoscopic view demonstrating a vascular lesion involving the left hypopharynx, with associated mass effect on the airway.

The patient remained stable without evidence of lesion progression (Table 1).

**Table 1:** Summary of clinical, imaging, procedural, and follow-up findings. BOT: base of the tongue, POD: postoperative day.

Parameter	Details
Age/sex	61-year-old female
Presenting symptoms	Progressive dysphagia, odynophagia, left-sided neck pain, globus sensation
Relevant history	Congestive heart failure, idiopathic cardiomyopathy, peripheral arterial disease, hypertension, dyslipidemia, tobacco use disorder, bladder tumor, aortic valve fibroblastoma, cardiac pacemaker
Initial imaging (February 2025 CT)	3.5 × 1.0 cm enhancing mass, left tongue base/lingual tonsil extending to hypopharynx; nodular calcifications of left aryepiglottic fold; concern for neoplasm
Interval imaging (July 2025 CT)	2.4 × 1.6 × 1.6 cm enhancing mass, left tongue base, glossotonsillar sulcus, lingual tonsil; extension to vallecula and piriform sinus; coarse bilateral paraglottic calcifications; narrowing of laryngeal inlet
Flexible laryngoscopy	Purplish, compressible vascular-appearing lesion, left aryepiglottic fold and tongue base; interval enlargement on follow-up; normal vocal cord mobility throughout
Procedure	Direct laryngoscopy with biopsy (Lindholm laryngoscope + rigid telescope); concurrent rigid esophagoscopy
Intraoperative findings	Hypervascular left supraglottic lesion (~1.5 cm) with calcification; nodular left BOT lesion (~1 cm); significant bleeding controlled with epinephrine-soaked pledgets
Histopathology	Venous malformation: disordered vascular channels (CD34/CD31/SMA+), intraluminal thrombi, hemosiderin deposition, phleboliths with ossification, chronic inflammation. No dysplasia or malignancy. Congo red negative
Postoperative course	Discharged POD 1; soft diet; resumed home medications
Follow-up	~6 weeks post-procedure; magic mouthwash for symptomatic (pain) relief; residual non-obstructive vascular lesion on flexible endoscopy; stable without progression

## Discussion

Venous malformations of the hypopharynx are rare and may present with nonspecific symptoms, including dysphagia, odynophagia, and globus sensation, which often lead to delayed diagnosis [2,3]. The deep location of the hypopharynx and its proximity to critical structures, including the aryepiglottic folds, piriform sinuses, and laryngeal inlet, further complicate clinical evaluation. In this case, the enhancing lesion with coarse calcifications on CT and compressible, purplish appearance on endoscopy was characteristic of a venous malformation, although preoperative cross-sectional confirmation was limited by the absence of MRI due to the patient's implanted cardiac pacemaker.

Imaging plays a key role in differentiating vascular malformations from other hypopharyngeal lesions. On contrast-enhanced CT, venous malformations typically demonstrate slow, heterogeneous enhancement and may contain phleboliths, which are highly suggestive of a low-flow lesion [3,5,6]. Calcifications and lobulated enhancing soft tissue reflect the chronic nature of the process. In contrast, squamous cell carcinoma, the most common neoplastic mimic, typically appears as an irregular, infiltrative mass without calcifications. Lymphatic malformations are generally cystic with minimal enhancement, while paragangliomas demonstrate intense arterial-phase enhancement with characteristic flow voids on MRI. MRI with T2-weighted sequences and gadolinium enhancement is considered the gold standard for characterizing vascular malformations and would ideally be obtained when feasible. In this case, CT and endoscopic findings were sufficient to raise a strong suspicion for a venous malformation and guide operative planning.

Compared with previously reported cases of hypopharyngeal venous malformations, this lesion demonstrated unusually extensive involvement spanning the tongue base, aryepiglottic fold, false vocal fold, vallecula, and piriform sinus [4,5]. The prominent bilateral paraglottic calcifications and phleboliths with ossification on histopathology indicate a longstanding lesion, consistent with the observation that venous malformations may remain clinically silent for years before becoming symptomatic [2]. Vahabzadeh-Hagh et al. similarly described a hypopharyngeal venous malformation presenting with foreign body sensation and dysphagia, confirmed on MRI and managed conservatively, reinforcing that symptom profile alone cannot reliably distinguish these lesions from malignancy [5]. In this case, the absence of MRI required greater reliance on CT and endoscopic features, highlighting the diagnostic value of calcifications and compressibility when advanced imaging is unavailable.

Histopathological findings confirmed a venous malformation demonstrating disorganized vascular channels with intraluminal thrombi, hemosiderin deposition, phleboliths with ossification, and positive immunohistochemical staining for CD34, CD31, and smooth muscle actin. These features support the diagnosis of a chronic low-flow vascular lesion and exclude malignancy.

Management of hypopharyngeal venous malformations is individualized based on lesion extent and symptom severity. Observation may be appropriate for asymptomatic or stable lesions, while intervention is reserved for symptomatic or progressive disease. Surgical excision or laser therapy may be considered for accessible lesions, whereas sclerotherapy is often preferred for deeper or more extensive involvement [3,7-10]. In this case, a biopsy was performed for diagnostic confirmation, given the uncertainty of imaging findings and the clinical concern for malignancy. Despite the vascular nature of the lesion and associated bleeding, the procedure was completed safely with appropriate hemostatic measures. The patient was discharged on postoperative day 1 without complications. At the six-week follow-up, she was managed with analgesics for symptomatic relief and remains stable. Long-term management options, including sclerotherapy, will be considered if symptoms progress.

This case highlights the importance of recognizing imaging features suggestive of a vascular malformation, particularly calcifications, heterogeneous enhancement, and compressibility on endoscopy, to avoid misdiagnosis as malignancy. It also demonstrates that biopsy of hypopharyngeal vascular lesions can be performed safely with appropriate precautions.

## Conclusions

Hypopharyngeal venous malformations are rare lesions that may mimic malignancy and present with dysphagia, odynophagia, or airway symptoms. Accurate diagnosis requires correlation of imaging findings, including characteristic calcifications and heterogeneous enhancement on CT, with endoscopic features such as compressibility and bluish discoloration, and ultimately histopathological confirmation. When MRI is contraindicated, CT and flexible laryngoscopy remain valuable diagnostic tools. This case highlights the importance of including vascular malformations in the differential diagnosis of hypopharyngeal masses and demonstrates that biopsy can be performed safely with appropriate planning. Conservative management with surveillance and symptomatic therapy is appropriate for non-obstructive lesions, with interventional options reserved for progressive disease.

## References

[1] Janardhan HP, Saheera S, Jung R, Trivedi CM (2021). Vascular and lymphatic malformations: perspectives from human and vertebrate studies. Circ Res.

[2] Buethe MG, Bayliss SJ, Shmuylovich L (2022). Venous malformations: a journey through their multifaceted clinical presentations. Dermatol Clin.

[3] Gallant SC, Chewning RH, Orbach DB, Trenor CC 3rd, Cunningham MJ (2021). Contemporary management of vascular anomalies of the head and neck-part 1: vascular malformations: a review. JAMA Otolaryngol Head Neck Surg.

[4] Roehm CE, Chelius DC, Larrier D, Hicks MJ, Albright JT, Valdez TA (2011). Postcricoid vascular lesions: histopathological and immunohistochemical diagnosis. Laryngoscope.

[5] Vahabzadeh-Hagh AM, Sepahdari AR, Fitter J, Abemayor E (2016). Hypopharyngeal venous malformation presenting with foreign body sensation and dysphagia. Am J Otolaryngol.

[6] Azizinik F, Javadi S, Khorasanizadeh F, Katouli FS, Majidazar R (2025). Vascular lesions of head and neck region: a pictorial review. Eur J Radiol.

[7] Klosterman T, O TM (2018). The management of vascular malformations of the airway: natural history, investigations, medical, surgical and radiological management. Otolaryngol Clin North Am.

[8] Alsuwailem A, Myer CM 4th, Chaudry G (2020). Vascular anomalies of the head and neck. Semin Pediatr Surg.

[9] Seront E, Vikkula M, Boon LM (2018). Venous malformations of the head and neck. Otolaryngol Clin North Am.

[10] Bertino F, Trofimova AV, Gilyard SN, Hawkins CM (2021). Vascular anomalies of the head and neck: diagnosis and treatment. Pediatr Radiol.

